# Rocks, teeth, and tools: New insights into early Neanderthal mobility strategies in South-Eastern France from lithic reconstructions and strontium isotope analysis

**DOI:** 10.1371/journal.pone.0214925

**Published:** 2019-04-03

**Authors:** Marie-Hélène Moncel, Paul Fernandes, Malte Willmes, Hannah James, Rainer Grün

**Affiliations:** 1 UMR 7194, CNRS, Institut de Paléontologie Humaine, National Museum of Natural History, 1 rue René Panhard, Paris, France; 2 SARL Paléotime, Villard-de-Lans, France; 3 UMR PACEA, CNRS, University of Bordeaux, CS, Pessac, France; 4 Department of Human of Evolution, Max Planck Institute for Evolutionary Anthropology, Leipzig, Germany; 5 Department of Wildlife, Fish, & Conservation Biology, University of California Davis, CA, United States of America; 6 Research School of Earth Sciences, The Australian National University, Canberra ACT, Australia; 7 Research Centre for Human Evolution, Griffith University, Nathan QLD, Australia; Max Planck Institute for the Science of Human History, GERMANY

## Abstract

Neanderthals had complex land use patterns, adapting to diversified landscapes and climates. Over the past decade, considerable progress has been made in reconstructing the chronology, land use and subsistence patterns, and occupation types of sites in the Rhône Valley, southeast France. In this study, Neanderthal mobility at the site of Payre is investigated by combining information from lithic procurement analysis (“chaîne evolutive” and “chaîne opératoire” concepts) and strontium isotope analysis of teeth (childhood foraging area), from two units (F and G). Both units date to the transition from Marine Isotope Stage (MIS) 8 to MIS 7, and show similar environmental conditions, but represent contrasting occupation durations. Level Gb (unit G) represents a long-term year-round use, in contrast to short-term seasonal use of the cave in level Fb (unit F). For both levels, lithic material and food were generally collected from a local to semi-local region. However, in level Gb, lithic materials were mainly collected from colluviums and food collected in the valley, whereas in level Fb, lithic procurement focused primarily on alluvial deposits and food was collected from higher elevation plateaus. These procurement or exchange patterns might be related to flint availability, knapping advantages of alluvial flint or occupation duration. The site of Payre is located in a flint rich circulation corridor and the movement of groups or exchanges between groups were organized along a north-south axis on the plateaus or towards the east following the river. The ridges were widely used as they are rich in flint, whereas the Rhône Valley is not an important source of lithic raw materials. Compared to other western European Middle Palaeolithic sites, these results indicate that procurement strategies have a moderate link with occupation types and duration, and with lithic technology. The Sr isotope ratios broadly match the proposed foraging areas, with the Rhône Valley being predominantly used in unit G and the ridges and limestone plateaus in unit F. While lithic reconstructions and childhood foraging are not directly related this suggests that the three analysed Neanderthals spend their childhood in the same general area and supports the idea of mobile Neanderthals in the Rhône Valley and neighbouring higher elevation plateaus. The combination of reconstructing lithic raw material sources, provisioning strategies, and strontium isotope analyses provides new details on how Neanderthals at Payre practised land use and mobility in the Early Middle Palaeolithic.

## Introduction

Neanderthal land use was complex and adapted to diversified landscapes, diverse climates and raw material sources (lithics and food resources). Current mobility models derived from ethnoarchaeological studies have shown that Neanderthals adapted their behaviour to the environment, alternating short and long-term occupations, described as base camps and/or satellite camps [1–14]. In south-eastern France, numerous Neanderthal sites link the medium-altitude regions of the Massif Central and Alpine foothills with the Rhône Valley. Over the past decade, considerable progress has been made in reconstructing the chronology, land use, subsistence and occupation patterns of these sites. Current models suggest complex occupation patterns, including long-term residential camps, as well as short-term regular hunting camps and brief stopover camps, showing that past populations may have been anticipating their land use requirements [15].

### The site of Payre

**Fig 1:**
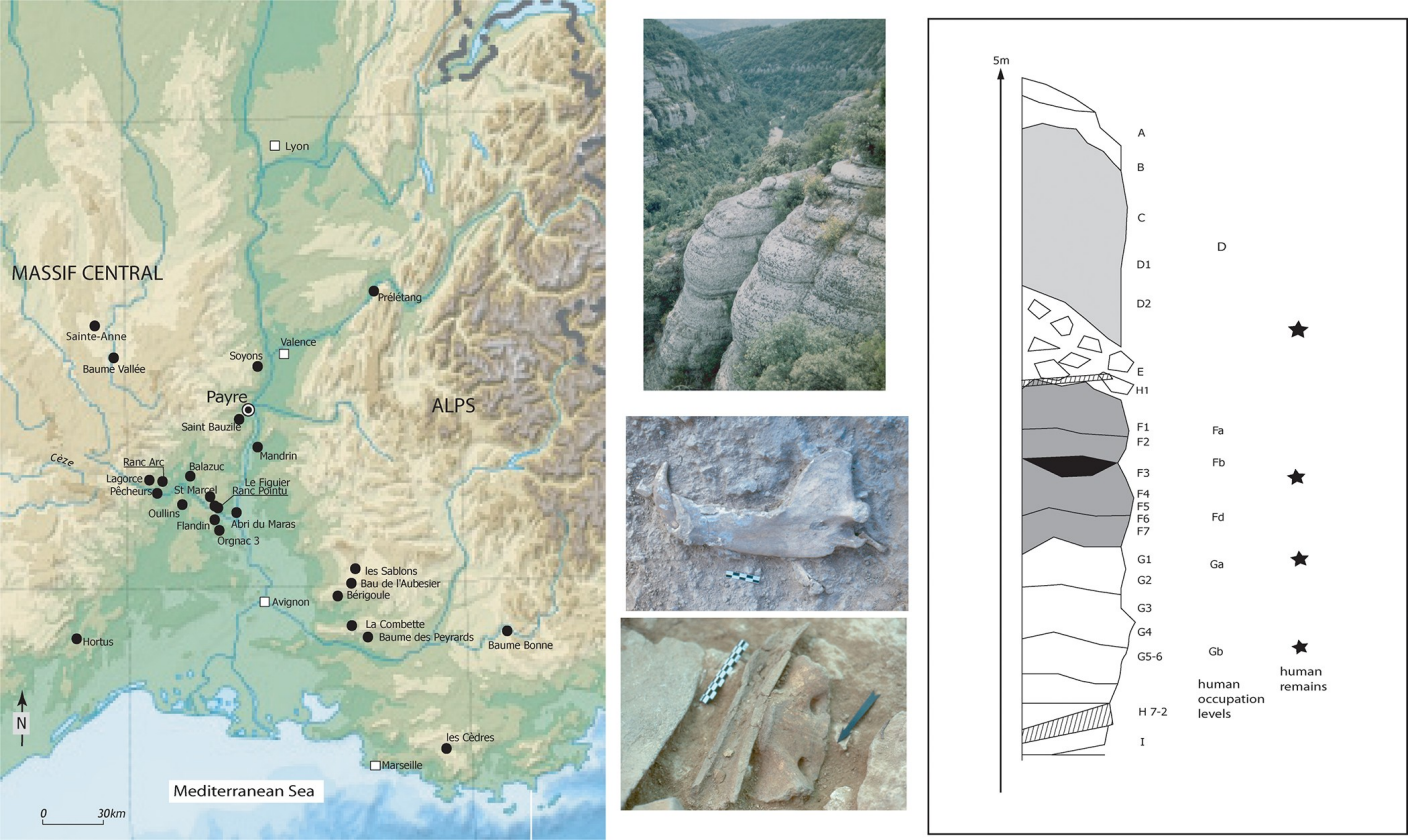
**Map of the location of Payre and other Middle Palaeolithic sites in south-eastern France (left) and site stratigraphy with the position of the human remains (right). In the middle, photos of the Payre Valley, *Ursus* mandible and fragments of ungulate bones**.

The archaeological site of Payre is a small cave above the confluence of the Rhône and Payre rivers in southeast France [15–20] (Fig 1). The five meter thick stratigraphic sequence at Payre consists of nine units (A-I), overlying the limestone bedrock. Human occupation is observed in eight levels during four units (C-G), with lithic material recovered throughout. The preservation of human remains at Payre is poor, with only several teeth and a fragment of a parietal bone from units C-G recovered from the entire site [21, 22, 23].

This study investigates lithics from level Fb (in unit F) and level Gb (in unit G) and three Neanderthal teeth from levels Fa, Ga, and Gb. All these levels have been dated to towards the end of MIS 8 and the beginning of MIS 7 by ESR and U-series dating analyses [24, 25]. Levels Fa and Fb are two phases of occupation at the top of unit F. Level Fb is a 15–20 cm thick grey sediment lens (F3) with no limestone blocks, just below level Fa. Level Gb contains a dense concentration of artefacts related to two lenses (G4 and G5), which are 50–65 cm thick and contain many small limestone blocks. The excavation of levels Gb and Fb extended over 50 m^2^ and 20 m^2^, respectively. Previous research at Payre on the faunal remains, cut marks, ungulate microwear patterns, and carbon and nitrogen isotopic analysis [15, 16, 26–36], all suggest a change in occupation patterns: Gb with long-term year-round use of the cave in contrast to short-term seasonal specific use (mainly in autumn) of the cave in level Fb. Furthermore, carbon and oxygen isotopic analysis suggests that the valley was exploited more during the long-term occupations of unit G, while the plateau above the site was more intensively used during the short-term occupation (for the four phases of occupation of unit F) [33].

Similarities in the faunal assemblage exist between levels Gb and Fb. The ungulate spectrum is mainly composed of red deer (*Cervus elaphus*), horses *(Equus mosbachensis*, bovines (*Bos primigenius* and *Bison priscus*) and rhinoceros (*Dicerorhinus hemitoechus* and *D*. *kirchbergensis*). Carnivores (*Canis lupus*, *Crocuta spelaea* and *Panthera* (Leo) *spelaea)* are especially numerous in unit F, suggesting that hominid occupations of the cave alternated with carnivore denning. Among them, *Ursus spelaeus* is predominant [16, 28]. The anatomical proportions of ungulates and location of anthropogenic marks indicate primary butchery activities for cervids and secondary butchery activities for bovines, horses and rhinoceros [16]. The occurrence of rhinoceros remains is considered to be mainly due to scavenging or occasional hunting in the Rhône Valley swamps at the foot of the cave [16]. These studies also suggest that in level Gb faunal remains were mainly accumulated by humans, whereas in level Fb carnivores played a large role in the consumption of carcasses [16]. In level Gb, mortality curves indicate hunting all through the year, while in level Fb hunting occurred more frequently in autumn [16]. Lithic residue and use-wear analysis shows evidence for fish processing and the use of avian resources in units F and D [35]. Burnt bones throughout the whole sequence provide evidence for the use of fire, with the possible use of bones as combustibles. The faunal and micro-faunal remains indicate a similar environment in both Gb and Fb: a cool climate and varying biotopes, including forests, wooded prairie, steep rocky slopes, as well as open steppe environments [29].

### Understanding land use and mobility at Payre

Studies of lithic raw materials are instrumental in determining land use and resource utilization. The lithic material recovered from these levels is attributed to the Early Middle Palaeolithic, with discoidal and orthogonal core technologies on flint present, and the toolkit mainly consists of scrapers and points [37]. Previous analyses of heavy-duty tools, including bifaces and pebble tools, show that they were made *in situ* and close to the site on local quartzite, limestone and basalt [31]. Most studies concentrate on the geologic composition of the flint material, but the technique used in this study also investigates the source of the lithic material in the landscape. This concept of the *chaîne evolutive* explores the underlying geology, identifies the accessible outcrops, and assesses the different stages of artefact production [38, 39]. The study of the technological stages, use, and disposal of artefacts (*chaîne opératoire)*, is applied alongside the *chaîne evolutive* [40–59]. If the assemblage is not disturbed by subsequent occupation or diagenetic processes, artefacts made from stone on site should be represented by remnants of each step of the production sequence. Identifying the procurement sites of these raw materials provides a ‘foraging radius’ for the population. The presence of artefacts made of non-local stone suggests human mobility or exchange [43].

In order to understand human mobility at the site strontium (Sr) isotopic analysis was undertaken. Strontium isotope ratios (^87^Sr/^86^Sr) vary between different geological regions depending on their age and composition, due to the radioactive decay of ^87^Rb by the emission of a negative *β*-particle with a half-life of ~4.88 x 10^10^ years to ^87^Sr [60]. The ^87^Sr/^86^Sr isotope signature of a region is mainly influenced by the underlying geology but can be augmented by additional sources of strontium from atmospheric deposition (precipitation, sea spray, dust) and exogenic surface deposits (loess, peat) [61–67]. Humans and animals incorporate strontium from their diet in their dental and skeletal tissues [68, 69], where it substitutes for calcium. ^87^Sr/^86^Sr isotope ratios of dental remains reflect the average isotope ratios of food intake and drinking water during childhood when the teeth were formed e.g. [61, 62, 66, 68]. Consequently, ^87^Sr/^86^Sr isotope ratios of permanent teeth can be used to assess mobility from a childhood residence area to a later residence area, across geologically different terrains. For hunter-gatherers, this becomes more complicated as the prey also move across the landscape, averaging the ^87^Sr/^86^Sr isotope variability of different geologic units. In this case, strontium isotopes can be used to draw broad scale inferences, but small-scale mobility may remain undetected. A general limitation of this method is that geographically distant areas can have similar or overlapping ^87^Sr/^86^Sr compositions.

This study will categorize the *chaîne opératoire* and *chaîne evolutive* of lithic artefacts found in levels Fb and Gb, identifying the origin of the raw materials used in artefact production at Payre. This lithic “foraging radius” will be combined with human childhood mobility data from ^87^Sr/^86^Sr isotope analyses of three teeth from Payre levels Fa, Ga, and Gb, to investigate mobility of early Neanderthals.

## Materials and methods

No permits were required for the described study.

### Composition of the lithic components of levels Fb and Gb

The artefact assemblage of level Fb is composed of 777 artefacts made predominantly from flint (over 93%), with small numbers of basalt, quartz, quartzite and limestone artefacts. The basalt artefacts consist of whole and broken pebbles, flakes and debris, the quartz artefacts are flakes and debris, and quartzite and limestone artefacts only consist of flakes. Flint artefacts are cores and flakes, with the entire flaking sequence present at the site.

Level Gb yielded 620 artefacts made predominantly of flint (over 92%), with small amounts of basalt, quartz, quartzite and limestone artefacts [16, 31] (Table 1). The basalt artefacts were brought to the site mainly as whole and broken pebbles or flakes. Quartz artefacts are all flakes, quartzite artefacts are flakes with one large tool, and limestone is rare, consisting of just one pebble and one flake. Flint artefacts consist of cores and flakes, with evidence of the whole flaking process.

**Table 1:** Lithic assemblage of levels Fb and Gb by raw materials at Payre.

Lithic type	Whole pebbles	Broken pebbles	Pebble- tools	Cores	Flakes (> 15 mm)	Micro flakes (< 15 mm)	Large tools	Total
**Level Fb**								
Basalt	5	1	1		17			24
Quartz					23			23
Quartzite					4			4
Limestone					2			2
Flint				7	167	549	1	724
**Level Gb**								
Basalt	10	6	6	1	1		1	25
Quartz					13			13
Quartzite					4		1	5
Limestone		1			1			2
Flint				10	515	48	2	575

### Reconstructing the *chaîne evolutive* and the *chaîne opératoire*

The concept of the *chaîne evolutive* describes the relationships between the environment and human behaviour in resource gathering and production [45–49]. In order to compare geological samples to archaeological artefacts, we characterized the mineral phases [50], analysed the different stages of artefact transformation steps [51, 52], and examined the artefact surface [53–57]. This resulted in the identification of the ‘genetic type’ (geological formation) of each lithic artefact and the specific details and transformation stages identify a ‘gitologic type’ (subtype or geological outcrop). The naming of artefact types from Payre consists of the initial of the first analyst (here F for Fernandes [39]), then the number of the genetic type from Payre, followed by a number indicating the gitologic type. In order to determine the sources of raw materials from artefacts, a lithothèque, or atlas of geological types and outcrops in the region, which encompasses the middle Rhône Valley between Valence in the north and Orange in the south, was developed based on ~200 geological samples. All samples were examined under a stereomicroscope, and thin sections of geological and archaeological samples were observed with a polarizing microscope and Scanning Electron Microscope (SEM). The *chaîne opératoire* was reconstructed for each artefact type by recording knapping, shaping and retouching modes to identify the techniques applied to each raw material [40, 41, 42, 58, 59].

### Strontium isotope analysis

Three Neanderthal teeth (Table 2) were selected from a complementary study on advancing analytical methods and the details are described in Willmes et al [70, 71]. For strontium isotope analysis micro-drilling followed by thermal ionisation mass spectrometry (TIMS) was used. In brief, a 0.3 mm drill bit was used to extract ~0.5 mg of sample material, which then underwent a weak acetic acid leach to remove possible residual contamination. The samples underwent ion exchange chromatography to isolate Sr from other elements using a micro column set filled with the Eichrom Sr specific resin. A drop of diluted phosphoric acid was added to each sample before loading onto rhenium filaments with a tantalum fluoride activator. Samples were measured on the TRITON *Plus* thermal ionisation mass spectrometer (TIMS) at the Research School of Earth Sciences, ANU. Data reduction includes a Rb correction, exponential mass bias correction (using the internal ^86^Sr/^88^Sr ratio of 0.1194) and 2σ outlier rejection. Total procedural blank levels for the human samples are below 100 pg Sr. Long-term measurements of the Sr carbonate standard SRM987 (National Institute of Standards and Technology) gave an average ^87^Sr/^86^Sr value of 0.71023 ± 2 (n = 99, 2σ) on the TIMS. This is consistent with the original certified ^87^Sr/^86^Sr isotope value of 0.71034±26 [72] and the more commonly quoted accepted value of 0.71025 ± 1 [73, 74, 75].

**Table 2:** Neanderthal teeth analysed from Payre in this study.

Sample ID	Level	Square	n°	X	Y	Z	Tooth type	Discovery year
336	Fa	I6	336	37	96	244	M1 lower right	2002
654	Ga	N8	654	77	5	511	M1-2 upper left	1999
6	Gb	Q8	6	40	60	530	M1-2 lower left	1994

In order to map the baseline bioavailable ^87^Sr/^86^Sr isotope variation for the region surrounding Payre, soil leachates and plant samples from the IRHUM (isotopic reconstruction of human migration) database were analysed [70, 75] and the ^87^Sr/^86^Sr isotope groups from Willmes et al [75] were used to cluster ^87^Sr/^86^Sr variability in the region based on surface geology. This provides a map of regional strontium isotope ranges and allows for the identification of mobility and land use between geologically distinct areas.

## Results

### Sourcing lithic artefacts from level Gb

**Fig 2:**
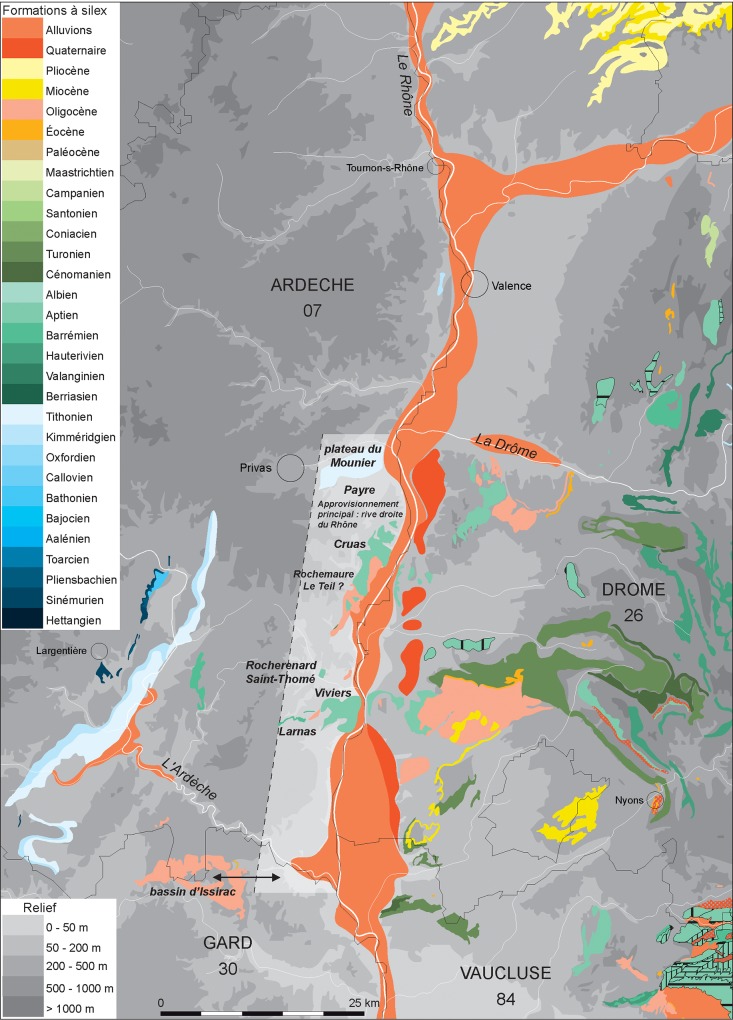
Map of the flint sources in level Gb at Payre.

**Fig 3:**
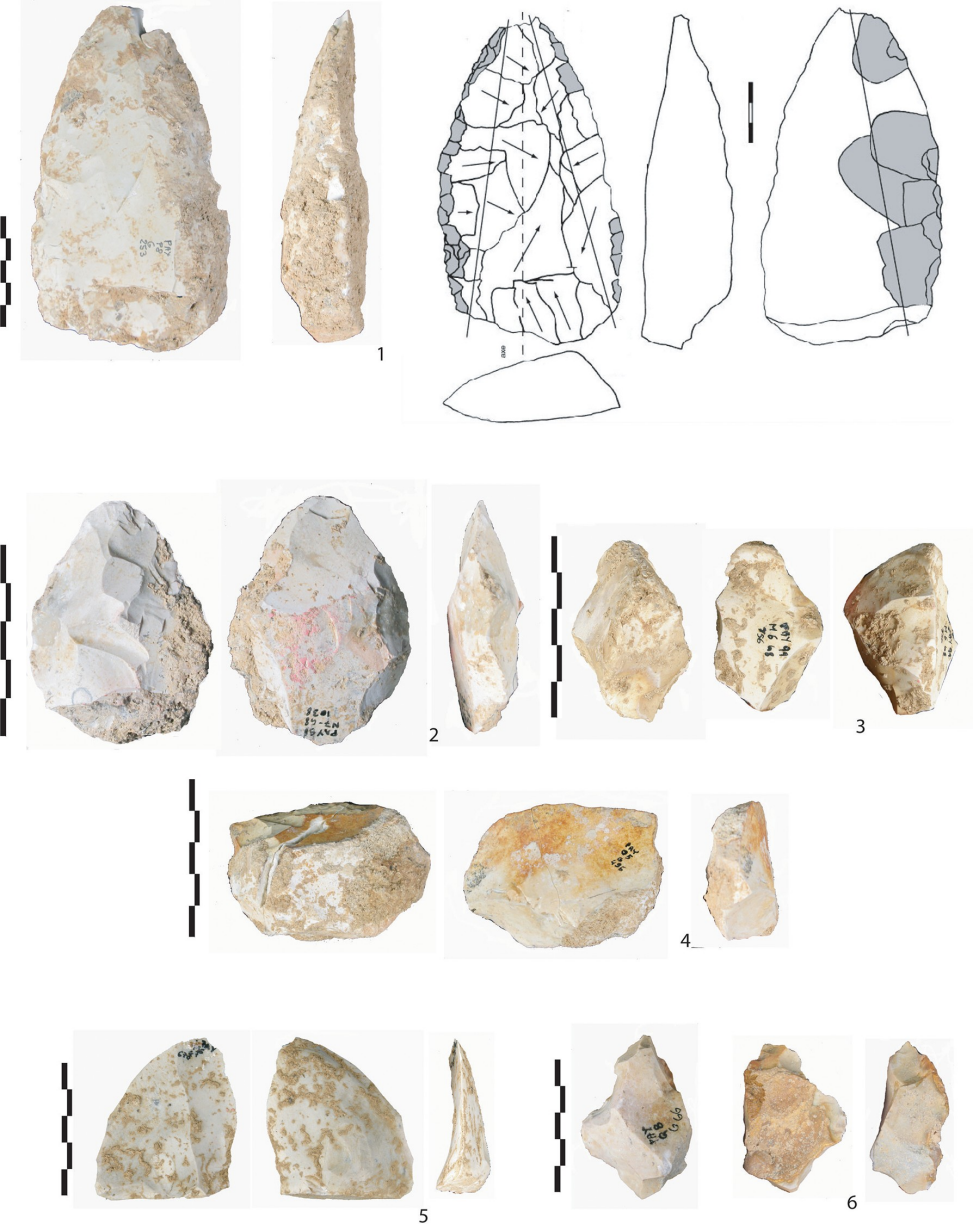
Overview of flint artefacts found in level Gb. (1) A biface tool on a large flake (F33, <30km), (2) biface tool on a small flake (F14, local), (3) abrupt retouch (“museau-type”) on a thick flake (F14, local), (4) a transversal scraper (F14, local), (5) unretouched flake (F34, semi-local), and (6) Bec-borers (F14, local).

A total of 575 lithic artefacts from nine genetic types were analysed, revised from [39, 76, 77, 78] and seventeen different procurement sites were identified, most of which are colluviums near primary formations (see the primary formations, Fig 2). The genetic types for these artefacts are described in Table 3. The four most common flint types in level Gb show differing procurement strategies. Type F14 (with spicules) consists of Upper Barremian flint collected as fragments of nodules or slabs on the Cruas Plateau on the right bank of the Rhône River, with some elements gathered from colluviums and alluvial deposits. Type F14 (bis), a Barremo-Bedoulian flint, was generally gathered as fragments of nodules on the right bank of the Rhône River. Type F34 flint, Bedoulian or Upper Barremain facies, was gathered as nodule fragments on the Cruas Plateau, and as pebbles in the rivers near Payre. Flint type F33, an Upper Barremian facies, was collected as pebbles or nodule fragments either from the river system at the base of the Bayne Plateau or on the Teil Plateau 30 km south of Payre. The rarest flint types also show differing procurement strategies. Type F120, a Tithonic flint, was collected as fractured fragments near Payre, types F124 and F127, which are Cenozoic age flints, were collected from the Issirac Basin south of Payre, and type F121 is of Alpine origin and Upper Cretaceous age and was collected from alluvial formations. Type F126 is marine flint of unknown origin and the one artefact of type F122 is of unknown origin. A total of 17 artefacts could not be sourced but appear to be made of Barremo-Bedoulian flint (Fig 3).

**Table 3:** Types of flint for level Gb at Payre. The details for 183 flint pieces could not be determined.

Genetic types	F120	F14-F14(bis)	F34	F33	F124	F127	F121	F122	F126
**Gathering zones**	Local	Semi-local < 10 km		Sub-Regional 10–30 km	Regional 30–60 km		Undetermined		
**Stratigraphy**	Tithonian	Barremian	Upper Barremian	Upper Barremian	Upper Eocene	Upper Eocene		Upper Cretaceous	
**Environment**	Marine	Marine			Lacustrine	Lacustrine		Marine	Marine
**Texture**	Mudstone	Wackestone	Mudstone	Wackestone	Mudstone	Mudstone, Wackestone	Mudstone	Wackestone	Wackestone
**Allochem**	Calpionellids	Spicule	Peloid, Spicule	Spicule	Gastropoda, Charophyta	Gastropoda Charophyta	Quartz geode	Spicule, Vegetal	Vegetal
**Type locality** **Or area**	Payre	Cruas plateau	Cruas plateau	Saint-Thomé, Bayne, Cruas	Issirac basin	Issirac basin			
**Secondary** **Stratigraphic** **position**		Oligocene to Pleistocene	Oligocene to Pleistocene	Oligocene to Pleistocene	Pleistocene	Pleistocene	Pleistocene	Pleistocene	Pleistocene
**Gitalogic** **types**	F120.2 F120.3	F14.1-F14.2 F14.3	F34.1 F34.2 F34.3	F33.1 F33.2 F33.3	F124.2 F124a.5	F127.2	F121.3	F122.1	F126.3
**Neo-cortex**	Colluvial, Alluvial	Alluvial, Colluvial sub primary	Alluvial, Colluvial sub primary	Alluvial, Colluvial sub primary	Colluvial	Colluvial	Subprimary	Alluvial	Alluvial
**Number**	**8**	**245**	**96**	**36**	**5**	**2**	**2**	**2**	**1**

Most of the observed material from level Gb was gathered from the Cruas Plateau, south of the site on the right bank of the Rhône. The flint was mainly gathered from the surface or the river network, at sites located between 5 and 15 km from Payre. The results indicate that groups mainly circulated on the plateaus along the right bank of the Rhône, along a N-S axis, up to about 60 km from the site, on the border of Gard. Flint was gathered from several types of superficial formations (colluviums, alluvial deposits and reworked formations linked to Oligocene conglomerate). This circulation is oriented towards the south, which contains abundant flint deposits of varying quality, as the site is at the northern limit of the Ardèche limestone. Based on our raw material analysis, the nearby Rhône Valley was used much less frequently, and materials from the nearby Alpine region are rare.

This diversity of provisioning sites can be considered from two angles. Neanderthals may have gathered flint during other subsistence activities. This could explain the relatively poor quality of certain nodules or pebbles, as they were gathered opportunistically based on shape criteria (block surfaces forming natural striking platforms) or appearance (fragments of rolled or non-rolled nodules). Alternatively, Neanderthals may have deliberately used different places for procuring flint material with the same macroscopic appearance. However, this would assume low flint availability, which is not the case in this region, although plant cover could have obscured sites. It is most likely that the diverse procurement sites observed were frequented during other activities.

Our identification of the *chaînes opératoires* for each genetic type of flint varies from complete to partial identification (Table 3). The most frequent flint types (F14, F34, F14bis, F33) come from a local to semi-local and sub-regional zone to ~30 km south of the site. Most of the production stages of the *chaîne opératoire* are represented for these flint types and an abundance of small-sized products (20–40 mm) were produced. The presence of cortical zones linked to colluviums and fluvial formations indicates sampling from varied deposits, but sampling from the Cruas plateau to the south of the site accounts for most of this flint material. Only artefacts of type F33, sourced from ~30 km from the site, were introduced as flakes, including the largest flake of the series.

For the rarest flint types (F120 to F127), the *chaînes opératoires* are only represented by several cortical or non-cortical flakes, indicating that these flints where brought to the site already worked (Table 4). Most of them measure between 20 and 60 mm. These flints appear rarely as tools (only a side scraper, a point, and a denticulate identified) and do not appear to be different to the flakes knapped on site. The observation of fluvial cortex and cortex from the colluviums indicates that these flints were gathered from diverse environments, either beside the Rhône River at the foot of the site (F120), or towards the south on the right bank. Two of these raw materials (F124, F127) show very similar mineralogical and micropaleontological aspects to the lacustrine flints from the Issaric basin and may have been collected ~60 km from the site, from a synclinal exploited later by the occupants of the nearby Abri des Pêcheurs site [38].

**Table 4:** Flint sources and technological features for level Gb (in numbers) at Payre (without badly preserved and largely broken artefacts.

	F120	F14	F14bis	F34	F33	F124	F127	F121	F122	F126
	Local on the site	Semi local < 10 km			Sub-Regional 10–30 km	Regional 30–60 km		?	?	?
Flakes <10–15 mm	1?	4	1	26	16					
15–40 mm	Cortical flakes 2 1 scraper	11 scrapers 10 points 1 notch 38 cortical and 67 non-cortical flakes	8 cortical and non-cortical flakes 1 scraper	39 cortical and non-cortical flakes 5 scrapers 1 point	1 cortical flake	3 cortical and non-cortical flakes		1 non-cortical flake		
40–60 mm		28 cortical and non-cortical flakes 8 scrapers 3 points	2 cortical and non-cortical flakes	3 cortical flakes 3 scrapers	6 cortical and non-cortical flakes 1 scraper			1 cortical flake		1 point
60–80 mm	1 denticulate	20 cortical and non-cortical flakes 6 scrapers 2 points		4 cortical and non-cortical flakes 1 scraper	3 cortical and non-cortical flakes 2 scrapers					
>80 mm		3 cortical and non-cortical flakes 1 biface on large flake 2 scrapers			1 cortical flake 1 biface					
debris	3	17	5	10	3	2	1		2	1
cores		7		4			1?			
Nodules left whole or partially flaked					2 (10–13 cm)					

The three most frequent types of flint (F14, F34 and F33) do not appear to have been collected and produced into tools in the same manner. Type F14 (Barremian) is the most abundant and was gathered from colluviums on the plateau. Retouched and non-retouched flakes are of varied dimensions and were mostly obtained from small fragments of nodules. Only a few large flakes with invasive retouch (semi-Quina) are present. Retouch is marginal and non-transforming. The only small biface is in this type of local sourced flint. Type F34 was gathered on the slopes of the Cruas plateau (reworked in colluviums from conglomerate deposits). The whole *chaîne opératoire* could have taken place on site (production and consumption of products). Type F33 comes from an intermediate zone between local and sub-regional provisioning areas (about 30 km away). However, it is represented by several large-sized retouched and non-retouched products. Tools were shaped on the largest of these flakes. The largest biface of the series is of type F33, as are two relatively good-quality tested nodules. This raw material could have been used to produce large flakes to be transformed into tools and flakes for knapping small flakes at the site. The large flakes, large tools, cores and the biface would thus have been imported. The presence of these nodules thus calls into question the model ruling out the transport of large-sized blocks over intermediate distances.

### Sourcing lithic artefacts from level Fb

**Fig 4:**
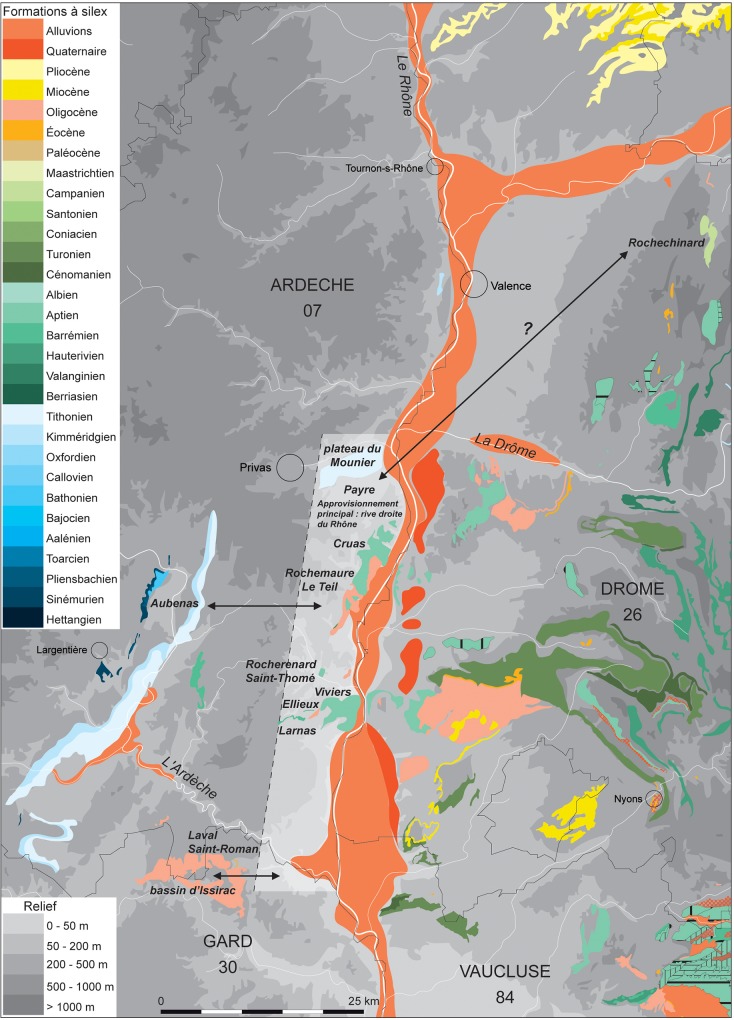
Map of the flint sources from level Fb at Payre.

A total of 198 flint artefacts from the level Fb assemblage were characterised. Most of the flint types present were sourced from alluvial deposits, making it impossible to identify the collection location of these materials (Fig 4, Table 5). Only 10% of the flints in level Fb are from colluvial deposits.

**Table 5:** Types of flint recovered from level Fb at Payre.

Types	F120	F14	F32	F35	F33	F165	F124	F215	F121	F122b
Potential gathering outcrops	Local	Right bank of the Rhône from Cruas	Right bank of the Rhône from Rochemaure, present at Saint-Marcel d'Ardèche	Right bank of the Rhône from Aubenas	Right bank of the Rhône from Viviers	Right bank of the Rhône from Saint Montan	indeterminate	Left bank of the Rhône?	Right bank of the Rhône from Rochemaure, present at Saint-Marcel d'Ardèche	indeterminate
Stratigraphy	Tithonian (cave)	Barremo-Bedoulian	Barremo-Bedoulian	Sinemurian	Barremo-Bedoulian	Lutetian	Ludian	Upper Turonian to Coniacian	Barremo-Bedoulian	Tertiary
Environment	Marine flint	Marine flint	Marine flint	Marine flint	Marine flint	Palustre flint	Lacustrine flint	Marine flint	Marine flint	Continental silicification
Geological formation	micritic limestone	limestone with spicules	limestone with flint	micritic limestone	limestone with flint	calcrete with Strophostoma and Planorbis	chalky limestone	limestone with bryozoa	limestone with flint	indeterminate
Geographical origin	Payre cliff-site	Cruas to Larnas area	Rochemaure-Le Teil	Aubenas	Saint-Thomé, Bois de Vallonge	Laval Saint-Roman, Ellieux	Issirac Bassin	Rochechinard	Viviers area Rocherenard	indeterminate
**Number of objects in level Fb**	2 objects	142 objects including 9 from Rochemaure	15 objects	4 objects	3 objects	2 objects	2 objects	6 objects	5 objects	2 objects
**Occurrence in level Gb**	yes	yes	indeterminate	no	yes	no	yes	no	yes	yes

The local and semi-local perimeter of the foraging radius is well represented by flint types F14, F32, and F120, with the latter collected at the foot of the Payre site. Flint type F14 with spicules is the most common and is represented by 104 objects, including nine from the base of the Rochemaure graben, previously described as type F34 [39]. They present marked similarities with the Barremo-Bedoulian flints (hemipelagic facies) from the north of the Cruas Plateau to Viviers, on the right bank of the Rhône. Their neocortex indicates a bipolar collection mode from the river network and from fossil alluvial formations. These groups are present in level Gb of Payre, and in the nearby sites of Abri des Pêcheurs, Abri du Maras, Barasses II (Balazuc) and at the open-air site of Saint-Bauzile (Andance). The type F32 artefact series contains three objects. The opaque and microcrystalline matrix presents some similarities with the flints derived from the limestones between Rochemaure and Teil and their neocortex indicates collection from the river network.

The regional perimeter (30–60 km) is represented by artefact types F35, F33 and F121. Type F35 includes five artefacts, which are similar to Sinemurian flint from the zone around Chapelle-sous-Aubenas. However, the neocortex indicates that they were gathered from the river network. This group is also used in Barasses II at the sites of Balazuc and Pêcheurs. Type F33 contains a single artefact with very similar structural and chromatic characteristics to the flints from the limestones in the sector of Bois de Vallonge (Saint-Thomé). The neocortex points to gathering from the river network. This type is also present in level Gb. Type F121 contains four artefacts. The Glomospira texture (with black patina) is very similar to the flints from the Bedoulian limestones of Rocherenard, to the northeast of Viviers. The neocortex indicates a gathering mode in the river network. This type is also present in level Gb of Payre.

Two flint types collected in a 60 km perimeter from Payre represent the outer extent of the foraging radius. Two type F165 artefacts have a similar texture to the Laval-Saint-Roman type calcretes and the neocortex indicates gathering from the river network. A single type F124 artefact is similar to the Ludian limestones from the Issirac basin. The neocortex indicates that it was gathered near a primary deposit. This type is also present in level Gb at Payre.

Finally, one type of flint is unknown (type F122b) and only one object has been identified. It presents some similarities with the Ludian limestone silicification from Issaric Basin. Due to the absence of neocortex, it is not possible to determine the formation type at the collection location. This type of flint was only found in level Gb at Payre and could have been collected east of the Rhône, perhaps providing evidence of a crossing of the Rhône River.

Four type F215 artefacts show a strong micropaleontological resemblance (presence of large bryozoan debris characteristic of the Coniacian) to the Upper Turonian to Coniacian limestones from the sector of Rochechinard (Drôme) located on the left bank of the Isère. Along with the F216 type present in level Gb, from more southerly Turonian limestones, these types represent a group of flint derived from Upper Cretaceous formations characteristic of the left bank of the Rhône. Their neocortex indicates gathering in the river network.

For level Fb, most of the artefacts were collected from a local or semi-local perimeter, with a few artefacts from over 30 km away from Payre. The lithological markers and the surface aspects point to a provisioning domain limited to the river network on the right bank of the Rhône, towards the east and the south, apart from the presence of flint from the Upper Cretaceous formations on the left bank of the Rhône (five objects). This type of behaviour is different to the diversity of sources used by the occupants in the underlying level Gb.

**Fig 5:**
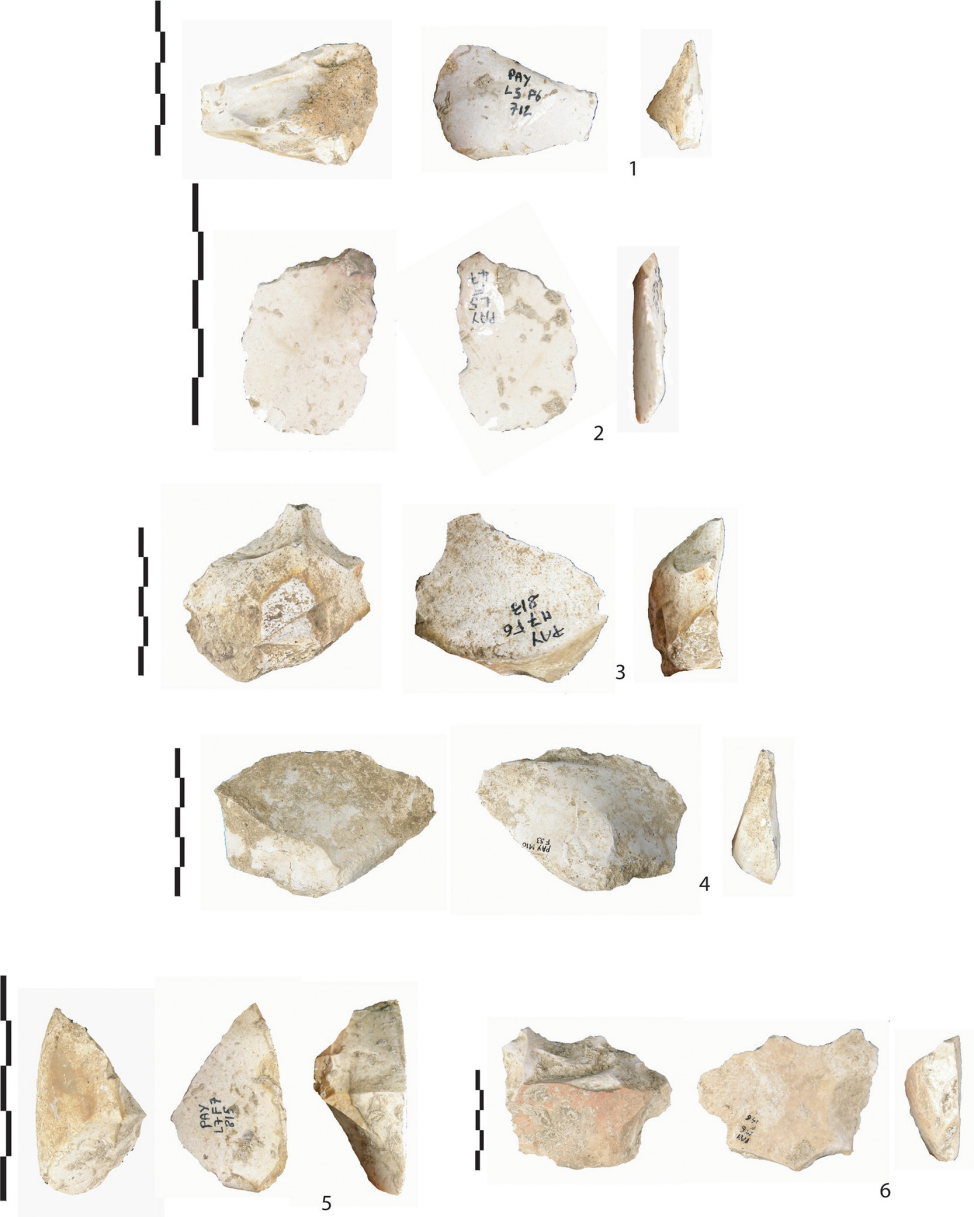
Overview of flint artefacts found in level Fb. (1) Unretouched flake with cortical zones (F14, local), (2) unretouched flake (F14, local), (3) Bec by Clactonian notches (F32, local and semi-local), (4) unretouched flake (F32, local and semi-local), (5) fragment of cortical flake (F215, East of the Rhône River), and (6) a denticulate (F32, local and semi-local).

Regarding technology and flint origin, three groups of objects can be identified (Fig 5, Tables 6 and 7). For the local flints (F14 and F32), all the stages of the *chaîne opératoire* are represented at Payre, associated cortical flakes, non-cortical flakes are represented, but small flakes of less than 15 mm, micro-flakes (retouch flakes) and rare cores are also present. These products are from the most frequent debitage method, i.e., unifacial discoid type debitage, conducted mainly on flakes. The few tools in the series are mainly on local flints (side scrapers, points, denticulates), including type F120, which is sourced locally. As for the more distant flint types, from sources located towards the east and the south in the regional sphere, the number of objects from all stages the production series is reduced. Only small flakes and flake fragments are present and most of these flakes are non-cortical and not retouched. The only flint type from the left bank of the Rhône is represented by non-cortical flakes and consists of two small flakes and flake fragments.

**Table 6:** Flint sources and types of artefacts for level Fb (in number) at Payre.

	F120	F14	F32	F35	F33	F165	F124	F215	F121	F122b
	Local	Semi-local and sub-regional 10–30 km		Regional perimeter 30–60 km				East of the Rhône		
Micro-flakes < 10 mm		7							1	
Flakes 10–15 mm from all phases		28	2	1				2	1	
Fragments	1	21	3	1	1		1	1		
Cortical flakes, first phases, with or without a back		8	1			1				1
Cortical flakes			1	1						
Backed flakes	1	5							1	1
Flakes with a cortical back		9	1							
Flakes with rare cortical patches (elongated, triangular, short)		15								
Non-cortical flakes		36	6	1	1	1	1	3	2	
Thick flakes from discoid cores or Quina-type		3			1					
Levallois flakes		3								
Kombewa flakes (various sizes)		4	1							
Cores		3								
Total		142	15	4	3	2	2	5	4	1

**Table 7:** Flint sources and flake-tools determined in level Fb at Payre.

	F14	F32	F35	F33	F122b
Origin	Semi-local and sub-regional 10–30 km		Regional 30–60 km		
Bec	2				
Denticulate	4				
Notch	3				
Multiple tool		1			
Point	1		1		
Scraper	8	1			
Denticulate scraper	3	2			1
Double scraper	4			1	

### Strontium isotope results

**Fig 6:**
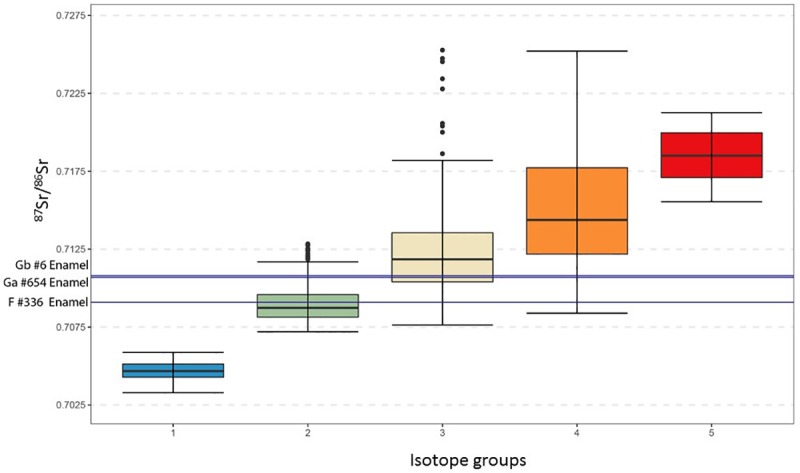
Box and Whisker plot of the isotope groups of France [76] with lines indicating the enamel ^87^Sr/^86^Sr values for the Payre samples.

**Fig 7:**
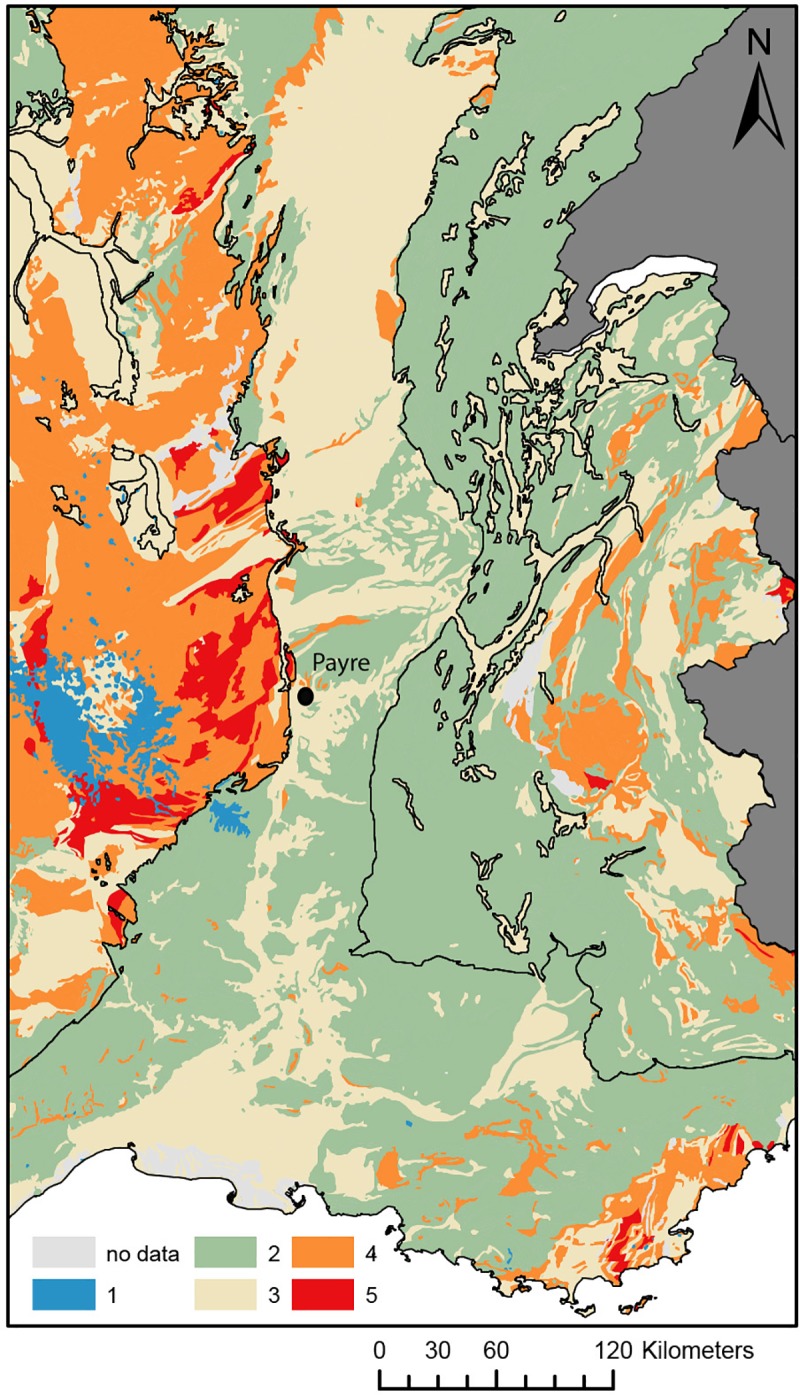
Strontium isotope groups based on the IRHUM database of ^87^Sr/^86^Sr isotope ratios of soil and plant samples for France [76].

Enamel and dentine of three Neanderthal teeth were analysed for strontium isotope ratios. ^87^Sr/^86^Sr of the enamel and dentine samples for each individual do not match, indicating that diagenetic overprint of the dentine occurred, altering the ^87^Sr/^86^Sr isotope ratio towards the local value (Table 8). The Neanderthals from unit G (sample #6, sample#654) have higher enamel ^87^Sr/^86^Sr isotope ratios of 0.71080±5 (2se) and 0.71069±2 (2se), respectively, compared to the Neanderthal from unit F (sample #336) with a value of 0.70909±3 (2se). The comparison of these values to the strontium isotope groups of France [75] excludes large areas of France as potential childhood residence areas, such as the volcanic units of the Massif Central (isotope group 1) and the granitic and metamorphic rock units (isotope group 4, 5) of the Massif Central. The closest match for the unit G Neanderthals is isotope group 3, which includes the sediments of the Rhône Valley and for unit F, isotope group 2, which includes the limestone units of the higher elevation plateaus. Some overlap exists between the isotope groups, and these results should be taken as a broad scale assignment rather than a classification to a single geological unit or geographic area (Figs 6 and 7).

**Table 8:** Strontium isotope results for the Neanderthal remains.

Sample ID	Material	^87^Sr/^86^Sr	2se	Level
336	dentine	0.70945	0.00006	Fa
336	enamel	0.70909	0.00003	Fa
6	dentine	0.70871	0.00008	Gb
6	enamel	0.71080	0.00005	Gb
654	dentine	0.70922	0.00043	Ga
654	enamel	0.71069	0.00028	Ga

## Discussion

### Lithic provisioning strategies at Payre

The analysis of lithic artefacts from levels Gb and Fb show, that in general, flint types are similar in these and outcrops in the local and semi-local and sub-regional region (from local to 30 km) were used during both periods of occupation. The flint sources for both levels are located to the east and south of Payre, on the low-altitude plateaus bordering the Rhône Valley or along the neighbouring river systems. The Rhône Valley was rarely used for flint procurement although small quantities of good-quality Alpine flint are present in the valley. Level Fb includes flint that may have been sourced from the east bank of the Rhône, potentially providing evidence of crossing the Rhône River by the inhabitants of Payre. However, based on present knowledge we cannot exclude procurement from the alluvial deposits of the Rhône during low water periods.

**Fig 8:**
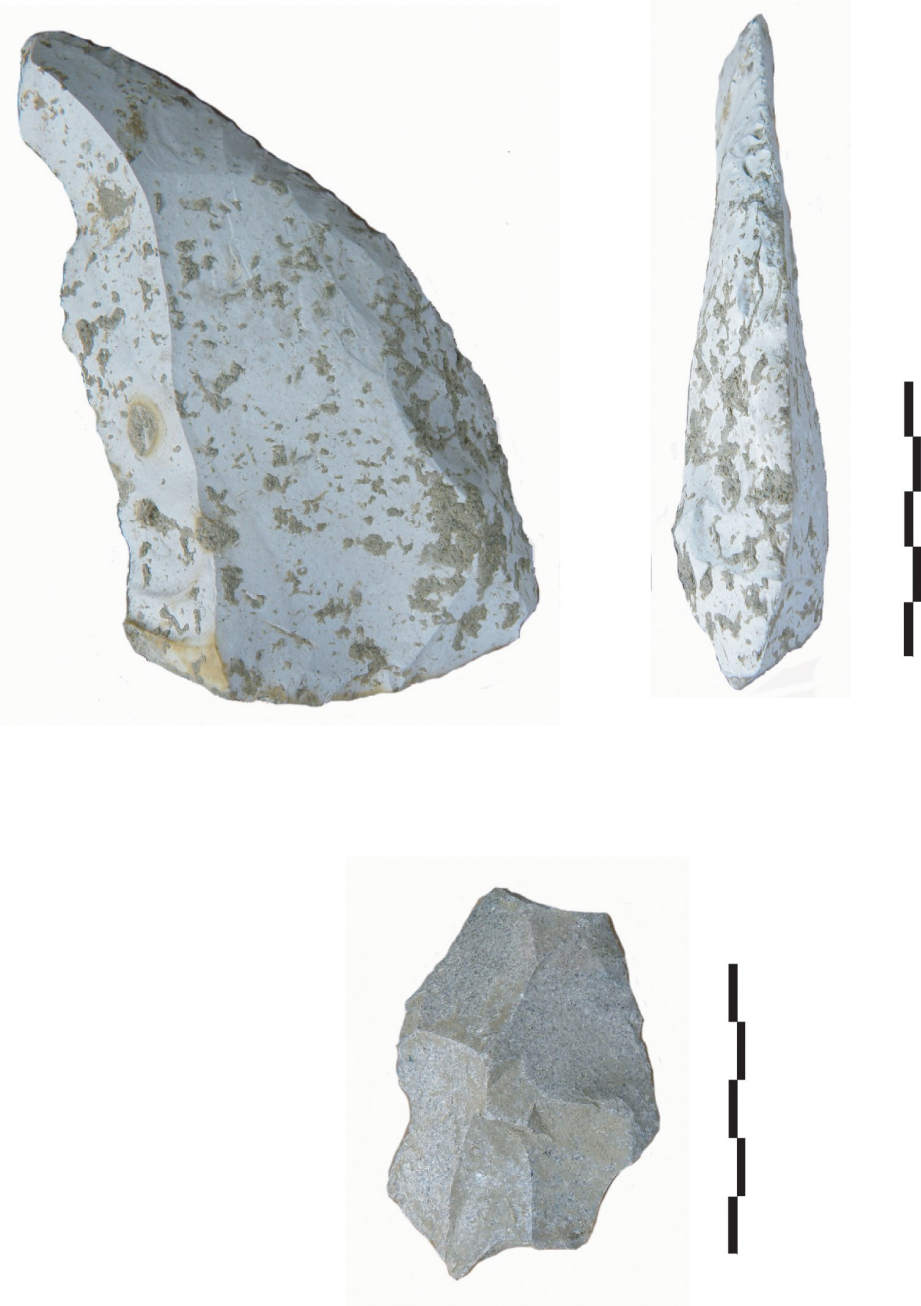
Flint Levallois flakes from level Fa at Payre.

The main difference between the lithic provisioning strategies during two occupation phases is that in level Gb, Neanderthals mostly collected raw materials from the colluviums, whereas in level Fb, procurement mainly focused on alluvial deposits. Different procurement patterns can result from many different factors including flint availability and quality, cultural differences, environmental changes, or occupation types and durations. For the two studied levels environmental conditions were similar and we find no evidence that flint availability or quality differed. Furthermore, micro-wear analysis and studies of residues on flint artefacts do not show any differences in the activities carried out on site, or any differences in the toolkits [35, 79, 80, 81]. Flint debitage methods, although similar in technique, are less diversified in level Fb (unifacial discoid debitage on flakes) compared to level Gb (unifacial, bifacial, multifacial debitage with different methods). Large pre-knapped flint flakes were brought to the site in both levels, from a local and semi-local zone. Note that in the other level Fa, above Fb, as in level Gb, four large Levallois flakes and a very large retouched Levallois point were also probably brought to the site from a semi-local perimeter, and from 60 km away respectively (Fig 8).

Lithic analysis at Payre shows that procurement strategies are directly linked to the type and duration of the occupation. The short-term occupants from level Fb gathered raw materials in valleys (alluvial deposits), whereas those occupying the cave for longer time periods (level Gb) moved about more on the plateaus (colluviums) and gathering flint during other activities. Regardless of occupation duration, we observe that: (1) flint provisioning occurs mainly in a local or semi-local perimeter, as does food sourcing and (2) flints from distant sources are not retouched, in either level. During periods of shorter occupation (level Fb), we observe that (1) distantly sourced flint types are more diversified, (2) tools are more frequent and are always made from local flints, (3) the number of small lithic pieces is higher, (4) the branching of the *chaîne opératoire* appears to be more marked with more flakes for debitage being brought to the cave, and (5) other rock types are used in smaller quantities. Artefact mobility appears to be higher for the short-term occupation of level Fb at Payre, with collection occurring mostly in valleys, perhaps during the movement between sites. The carbon and oxygen isotopic analysis of the faunal remains in the cave and one Neanderthal tooth [33, 34, 82] show that the occupants of unit G were using the Rhône Valley more often than those of level F, whose activities are more closely linked to the plateaus above the site.

### Land use and mobility on a regional scale

A comparison between Payre (levels Gb and Fb) and three more neighbouring sites (Saint-Bauzile, Abri des Pêcheurs, and Abri du Maras), using the same methodology, also suggests a link between the types and duration of occupation and raw material procurement patterns.

The site of Saint-Bauzile (Andance), located less than 1 km from Payre on a basaltic dome, has yielded remains of short-term open-air occupations dated to before MIS 5 [83, 84]. The main flint type (F14), derived from the Barremo-Bedoulian, was collected from colluvial and alluvial Oligocene conglomerates ~5 km from the site. Type F14 flint is associated with some semi-local Jurassic flint, silcrete (F120), and other regional flint. As at Payre, this open-air occupation shows preferential procurement on the plateau to the south. But in this case, procurement is concentrated in a single sector (the plateau of Barrès several km from the site), and is less diversified, suggesting opportunistic gathering on the way to the site.

The second comparison site of Abri des Pêcheurs is located to the west in the Chassezac Valley, a tributary of the Ardèche River. The site records evidence of brief and recurrent occupations by human groups (bivouacs) using the site as a refuge at the beginning of a severe cold climate phase in the region. Several lithic series, primarily made up of non-retouched flakes and cores in quartz (between 60 and 95% of the series), provide evidence of successive Middle Palaeolithic human occupations between the end of MIS 5 and MIS 4 [24, 26, 85, 86, 87]. These series only contain flakes and three cores in flint. The type of procurement observed is different to that identified in level Fb at Payre, even though occupation duration is similar. A wider diversity of flint types from alluvial deposits and colluviums from a larger perimeter extending in several directions is seen at Abri des Pêcheurs. This indicates toolkits may have circulated from site to site. Twenty-seven flint deposit types (representing 21 genetic types) have been identified. They correspond to many different gathering places; F34 and F14 type from Larnas zone (30 km from the site), from colluviums to the south and north of the site (F168), from the Barjac–Issirac basin around 20 km away (types F166, F167, F171, F179), the Laval-Saint-Roman zone 25 km from the site (F165 and F172), from the Lumachelle and Entrochal limestone less than 10 km south of the site (F35) (Tables A and B in S1 File).

A comparison of short-term occupations (Payre Fb, Abri des Pêcheurs, Saint-Bauzile), to levels 4.1 and 4.2 (beginning of MIS 3) from Abri du Maras, located further south, on the edges of the Ardèche River, shows that different provisioning strategies were seen for seasonal occupations, with Levallois-type series present at Abri du Maras. The provisioning perimeter is ~30 km with gathering occurring to the north and south of the site, on the plateaus. Pre-knapped blades, points and large flakes were imported to the site and complementary Levallois debitage on flake cores took place in the shelter [20]. This may suggest that the inhabitants of this site anticipated their needs, travelling in a territory extending from the northern to the southern plateaus. A total of 17 types of flint has been identified which indicates a restricted procurement area along the right bank of the Rhône [20].

Our petrographic and technological regional data indicate that procurement patterns differ, sometimes in relation to the duration and type of occupation, but not in relation to technological strategies, regardless of the age of the occupation. Through provisioning flint sources, we can reconstruct that the occupants of Payre collected flint from the same formations at the same *chaîne évolutive*, but in different locations. Our results indicate that the local and semi-local perimeter was systematically exploited for the collection of certain types of flint and food resources. The artefacts from the most distant sources, which are potentially the most mobile objects, are no more retouched than the others. This situation is similar to other regions rich in flint, such as the southwest of France, where the local network provides the most mobile objects [88, 89]. At Payre, in level Gb, the small biface is the most frequently used local flint type and the largest is in semi-local flint, which is also widely used. The other raw materials are sometimes knapped before being brought to the site and are represented by small flakes, large flakes or large shaped objects [31], indicating the circulation of objects in a local perimeter.

We observe two types of flint management based on the distance of raw material sources and the type of occupation in this region. In levels Gb and Fb at Payre and at the Saint-Bauzile site, large quantities of flint of diverse quality were gathered from the nearest deposits, although occupation durations differ. The provisioning area comprises many flint deposits, suggesting that flint was gathered during other subsistence activities. The presence of these flint deposits may partly account for the choice of the occupation zone.

At the site of Abri des Pêcheurs, the flint is sourced from many sectors, which correspond to a wider operating area. Most of the flint sources are located to the east of the site and on the edge of the Rhône Valley. But as at Payre in level Gb, none of the identified facies come from the other bank of the Rhône. Flints (including local flints) appear to be transported, and not knapped on site. The location of flint deposits is independent of the choice of habitat. The territory is vast but appears to be limited to the right bank of the Rhône. This could indicate a circulation area of human groups using the cave on an occasional basis.

Unlike present models, these examples show that occupation type can be linked to procurement type, proposing that relatively long-duration seasonal occupations are linked with mainly local procurement, with some flint from more distant sources (collected during subsistence activities or brought as a toolkit), and that occasional occupations are linked with local materials, but also flakes from a wider zone knapped outside the site.

The age of the sites and the environmental conditions cannot completely explain these strategies. Even if steppe-type plant cover during the Abri des Pêcheurs occupations, led to different types of subsistence and to the increased mobility of human groups. We are not able to explain why flint from the most distant sources was only left at sites with short-duration occupations. If this flint was moved from site to site as part of the toolkit, why does it not occur in longer duration occupations? Lastly, how can we measure the degree of mobility of the most abundant flint types found at all the sites? Comparisons can be extended to the southeast Massif Central plateaus of nearby Velay where contemporaneous occupations have also been investigated at Sainte-Anne I and Baume-Vallée (Haute-Loire). Although the geological conditions are not identical at Payre and Sainte-Anne I (level J1 dated from MIS 6 to 5), raw material procurement follows the same patterns at both sites. This shows that Neanderthals adapted to the geological context according to their needs and types of occupation [77–80, 90–92]. The same can be seen at Baume-Vallée, where seasonal occupations dated to MIS 4 show the same types of strategies and territorial occupation as at Payre and Sainte-Anne 1.

### Neanderthal land use

Bordes [93], Bordes et al. [94] and Binford [95] proposed a model of different types of sites having specific functions. Binford [1, 2, 3] and Kuhn [96] suggested that a relationship existed between raw materials, tool types, and occupation duration. At Payre, our results from flint procurement have led us to conclude that: (1) when hominins transported specialized tools from distant outcrops (distant sources and mobile tools indicate provisioning from distant outcrops) or used multifunctional and well-maintained tools, occupations were generally short-term, and (2) when requirements were met by bringing raw material from local outcrops to the site, along with some tools brought in by the group from other sites, occupations were relatively stable and of longer duration. The first case indicates high human mobility, a specialized camp, or raw material scarcity, while the second case shows occupations based on the exploitation of the site surroundings, with diverse activities taking place within a foraging radius. Strontium results indicates the childhood land use pattern of three individuals and shows they could have grown up near Payre. The long-distance flint present in level Gb can thus be attributed to exchanges between groups or high Neanderthal mobility on plateaus bordering the Rhône River.

These models tend to contrast (1) definite mobility by small groups with frequent seasonal movements in a local and semi-local area in relation to local resources and short-term occupations, such as task-specific locations or seasonal occupations with diverse activities, and (2) differential mobility with base camps or longer occupations by whole groups and satellite camps for specific activities by other task-groups with shorter occupations [3, 97–100].

These patterns may vary throughout the year and in different regions. In a single site, such as a cave or a rock shelter, different types of occupations can be observed over time [97–104]. For Middle Palaeolithic settlement organization in Europe, in the same region and environmental context, diversified subsistence strategies seem to have coexisted [89, 105, 106]. In south-western Europe, base camps and butchery sites exist in Crimea and south-eastern France, where a variation of the radiating mode is recorded [12, 27]. In many cases, it is not possible to distinguish a base camp from a seasonal camp or a hunting site. Subsistence activities and food consumption may have taken place together at relatively long-duration hunting sites [12, 15, 90, 91, 107, 108].

The use of a classic analytical method alongside the genetic and post-genetic history of flint shines a light on our understanding of raw material procurement. It enables us to accurately identify procurement sites and thus to assess Neanderthal land use at a given site. On the right bank of the Rhône Valley, which forms a vast circulation corridor, the geological context is rich in flint. The sites show that the circulation of human groups or exchanges between groups are organized on the limestone formations, along a south-north axis on the plateaus on the edge of the Rhône Valley, or towards the east, the northeast and the southeast, partly following the river system. Hominids preferentially used large quantities of the most accessible flints. The ridgelines were widely used as they are rich in flint, whereas the Rhône Valley does not seem to have been considered as a potential source. The distance to the furthest outcrops can be over 60 km, or less than 30 km, at the different sites. However, the circulation of materials collected from the periphery of the area is not characterized by more intensive use, unlike at other sites, like those in southwest France. The meaning of mobile objects and rocks rarely collected by Neanderthals thus remains difficult to explain. Mobile objects could represent mobile tool kits, *cf*. [11, 109–112].

Previous studies show links between the availability of local rocks and the types of occupations [88, 113–115], as in the south-eastern margins of the Massif Central. In Spain, for instance, in sites with short-term occupations, local stones are mainly associated with some long-distance retouched objects, such as Teixoneres during MIS 5–3 [116–120]. At Abric del Pastor [121], short occupations during MIS 5–4 indicate a radius of 15 km with some long-distance artefacts/preforms. At Las Callejuelas, at an altitude of 1,400 m [122], only local flint was used during specialized occupation in mountain zones. In most of the sites, local materials were predominantly used, even when they are of poor quality, and rocks from more distant outcrops (30–100 km away) are often present only in small quantities, regardless of technological behaviour and land use patterns, or the age of the occupation [12, 103, 123–130]. Neanderthals used the valleys as circulation routes or crossed interfluves [131–133].

More rarely, the type of core technology (expedient or requiring more cost-investment) influences logistics [134]. For example, at Abric Romani [135, 136], discoid technology is associated with low mobility, unlike Levallois technology in south-western France [88, 111]. For our sites, this is not the case. Distance from procurement zones is more dependent on the type and duration of the occupation, even though the predominant use of discoid technology at Payre is mainly associated with local procurement, whereas Levallois technology at Abri du Maras is associated with broader procurement for most of the materials. As Turq et al. [88] describe, we are in a “centrifugal” configuration for the most widely used local materials and a “centripetal” configuration for the rare objects from more distant outcrops. In Belgium [137–139], the technology and *chaines opératoires* are linked to the distance from raw material sources and Levallois débitage is more frequent when the flint is near the site. In Central Europe, the Micoquian indicates low raw material diversity (radiating model), while the Mousterian shows high raw material diversity (circulating model) [140].

### Neanderthal mobility

The extent of Neanderthal mobility is currently poorly understood. Lithic raw materials found at Neanderthal sites are often local, with only a small percentage from sites more than 5 km away, suggesting limited mobility during the occupation. Some exotic materials do occur at sites, suggesting considerable Neanderthal mobility at times, like the suggested mobility observed for *Homo heidelbergensis* [141, 142]. In a region with abundant flint resources where the same types are regularly gathered from different places, it is difficult to explain mobility patterns [143]. However, lithic materials can travel by exchange and thus are not a direct measurement of mobility. Zooarchaeological evidence from the Quina Mousterian deposits from the site of Jonzac (Charente-Maritime, France) indicates that Neanderthals hunted seasonally abundant reindeer in short-term hunting camps [144]. This would support the idea of Neanderthals as highly mobile hunter-gatherers. Strontium isotopic investigations of reindeer by Britton et al. [145] further support this interpretation, by showing that these reindeer were probably killed during the same hunting event. Migrating herbivores can be considered as an important prey species for Neanderthals and potentially exhibit complex and changing mobility patterns. This adds complexity when trying to infer Neanderthal childhood residence areas from strontium isotopes because a change in strontium isotope ratios might simply reflect the changing mobility pattern of the dominant food source. Richards et al. [146] first applied strontium isotope tracing to directly investigate the mobility of a Neanderthal individual from the site of Lakonis, in Greece. They argued that this individual spent its childhood in an area at least 20 km away from the site. However, due to potential problems with the analytical technique they used, as well as the lack of a strontium isotope baseline map for the area, their interpretation of the data has been disputed by Nowell and Horstwood [147] (see Richards et al. [148] for further discussion). At Payre, the strontium isotope results indicate a childhood foraging area that broadly matches the lithic reconstructions, with the Rhône Valley being predominantly used in unit G and the limestone plateaus in unit F. However, strontium isotope ratios in tooth enamel reflect only the childhood foraging area, and thus may be decoupled from lithics found in the same level. Nevertheless, our results support the idea of mobile Neanderthals in the Rhône Valley and neighbouring higher elevation plateaus.

## Conclusions

The combination of reconstructing lithic raw material sources, provisioning strategies, and strontium analyses provides new details on how Neanderthals at Payre practised land use and mobility in the Early Middle Palaeolithic. On the south-eastern fringes of the Massif Central, the diverse Middle Palaeolithic strategies show a moderate link with occupation types and duration, and with technology. The region contains abundant flint resources, which may explain permanent choices and behavioural patterns, the circulating mode on limestone formations, as well as the low intensity of artefact curation. Local materials are systematically used regardless of occupation duration in a region where no specialized butchery sites have yet been discovered. The higher diversity of flint types and thus of procurement sites during short occupations has yet to be explained, as has the abandonment of the small toolkit during these occupations. Local flint can circulate in the same way as objects from the regional perimeter, putting the current land use model into question. Strontium analyses of human teeth at Payre indicate food procurement during childhood occurred in a local to semi-local perimeter. While lithics and teeth are not directly related, these findings agree with the foraging radius suggested by the lithic analysis and suggest mobile Neanderthals in the Rhône Valley and on the surrounding higher elevation plateaus. Definitive data regarding circulation patterns, information about circulation directions, minimum distances travelled, possible exchanges and topographical constraints remain unclear. The reasons behind the choice of some raw materials also remain enigmatic as strategies may not be directly related to raw material accessibility and abundance. Further research is required in order to understand the cultural factors behind land use patterns and the choice and use of specific raw materials by Neanderthals.

## Supporting information

S1 File Table A. Types of flint used at Abri des Pêcheurs in the Middle Palaeolithic sequence (MIS 4), after [84]. Table B. Types of flint and products in the Middle Palaeolithic sequence at Abri des Pêcheurs.
(DOCX)
