# Supplementary material for: Rocks, teeth, and tools: New insights into early Neanderthal mobility strategies in South-Eastern France from lithic reconstructions and strontium isotope analysis
Source: PLoS One. 2019 Apr 3;14(4):e0214925. doi: 10.1371/journal.pone.0214925 (PMC6447223; doi:10.1371/journal.pone.0214925)
Supplement: S1 File — Table A. Types of flint used at Abri des Pêcheurs in the Middle Palaeolithic sequence (MIS 4), after [84]. Table B. Types of flint and products in the Middle Palaeolithic sequence at Abri des Pêcheurs. (DOCX) [file pone.0214925.s001.docx]

Suppl. Table A.

| Stratigraphic origin | Kimmeridgian | Carixian | Lutetian | Lutetaen | Ludian | Ludaen | Cenozoïc | Ludaen-Sannoisaen | Barremian | Bedoulian | Ludaen | Jurassic | Cenozoïc | Indeterm. | Indeterm. | Indeterm. | Indeterm. | Indeterm. | Indeterm. | Indeterm. | Indeterm. | Indeterm. |
| --- | --- | --- | --- | --- | --- | --- | --- | --- | --- | --- | --- | --- | --- | --- | --- | --- | --- | --- | --- | --- | --- | --- |
| *Distances* | *local* | *local* | *25 km* | *25km* | *25km* | *25 km* | *25 km* | *25 km*  *25 km* | *30 km* | *30 km* | *?*  *?* | *?* | *?*  *?* | *?* | *?* | *?* | *?* | *?* | *?* | *?* | *?*  *?* | *?* |
| Type n° | 168 | 35 | 165 | 172  172 | 167  167 | 171 | 166 | 179 | 14 | 34 | 169  169 | 170 | 273 | 173 | 174 | 175 | 176 | 177 | 178 | 181 | 210 | other |
| F11 to F14 | 1 |  | 1 |  |  |  |  |  | 1 | 2 | 1 | 2 |  | 1 | 1 |  |  |  |  |  |  | 1 |
| F15-16 | 1 |  |  |  |  |  |  |  |  |  |  |  |  |  |  |  |  |  |  |  |  |  |
| F17-F18 | 1 |  |  |  |  |  |  |  |  | 1 |  |  |  |  |  |  |  |  |  |  |  |  |
| F19 | 3 |  |  |  | 1 | 2 | 2 |  |  |  | 1 |  |  |  |  |  |  |  |  |  |  |  |
| F20 to F23 | 1 |  |  | 1 | 3 |  |  |  |  | 1 | 1 |  |  |  |  |  |  |  | 1 |  |  |  |
| F24 to F26 | 1 |  |  |  | 2 |  |  | 1 |  | 2 | 1 |  |  |  |  |  |  |  |  | 1 |  |  |
| sector 4  base of the sequence | 7 | 3 | 2 |  | 2 | 2 | 1 |  | 1 | 9 | 4 |  | 1 |  |  | 1 |  | 1 |  |  |  | 1 |
| Indeterminate | 12 | 2 | 16 | 2 | 9 | 12 | 1 | 1 | 8 | 26 | 1 | 4 | 1 | 1 | 1 |  | 2 | 4 | 2 |  | 1 | 1 |
| total | 27 | 5 | 19 | 3 | 17 | 16 | 4 | 2 | 10 | 41 | 9 | 6 | 2 | 2 | 2 | 1 | 2 | 5 | 3 | 1 | 1 | 3 |

Suppl. Table B.

| Stratigraphic origin | Kimmeridgian | Carixian | Lutetian | Lutetaen | Ludaen | Ludaen | Cenozoïc | Ludaen-Sannoisan | Barremian | Bedoulian | Ludaen | Jurassic | Cenozic | Indeterm. | Indeterm. | Indeterm. | Indeterm. | Indeterm. | Indeterm.. | Indeterm. |  | Indeterm. | Indeterm. | total |
| --- | --- | --- | --- | --- | --- | --- | --- | --- | --- | --- | --- | --- | --- | --- | --- | --- | --- | --- | --- | --- | --- | --- | --- | --- |
| *Distances* | *local* | *local* | *25 km* | *25km* | *25km* | *25 km* | *25 km* | *25 km*  *25 km* | *30 km* | *30 km* | *?*  *?* | *?* | *?*  *?* | *?* | *?* | *?* | *?* | *?* | *?* | *?* |  | *?*  *?* | *?* |  |
| Type n° | 168 | 35 | 165 | 172  172 | 167  167 | 171 | 166 | 179 | 14 | 34 | 169  169 | 170 | 273 | 173 | 174 | 175 | 176 | 177 | 178 | 181 |  | 210 | others |  |
| Flakes | 23 | 4 | 15 | 1 | 7 | 10 | 2 | 1 | 7 | 20 | 8 | 6 | 1 | 1 |  | 1 | 1 | 2 | 2 | 1 |  | 1 | 2 | 116 |
| Fragments | 1 |  | 4 | 2 | 3 | 2 |  | 1 |  | 6 | 1 |  |  |  |  |  |  | 2 |  |  |  |  | 1 | 23 |
| Elongated  products | 1 |  |  |  | 5 |  |  |  |  | 4 |  |  |  |  |  |  |  |  |  |  |  |  |  | 10 |
| cores |  |  |  |  |  |  |  |  |  | 1 |  |  |  |  | 1 |  |  |  | 1 |  |  |  |  | 3 |
| Retouched  flakes | 3 | 1 |  |  |  | 5 | 2 |  | 3 | 10 | 1 |  |  | 1 | 1 |  |  |  |  |  |  |  |  | 27 |
